# Evaluating the Hebrew version of the financial exploitation vulnerability scale

**DOI:** 10.1093/geront/gnag050

**Published:** 2026-04-17

**Authors:** Gali H Weissberger, Lisa Engel, S Duke Han, Peter Lichtenberg

**Affiliations:** Department of Social and Health Sciences, Bar-Ilan University, Ramat Gan, Israel; Department of Occupational Therapy, College of Rehabilitation Sciences, University of Manitoba, Winnipeg, Manitoba, Canada; Institute for Work and Health, Toronto, Ontario, Canada; Department of Psychology, USC Dornsife College of Letters, Arts, and Sciences, Los Angeles, California, United States; Institute of Gerontology, Wayne State University, Detroit, Michigan, United States

**Keywords:** Financial exploitation, Vulnerability, Reliability, Validity

## Abstract

**Background and Objectives:**

Assessing financial exploitation (FE) in older adults has proved challenging for various reasons, including low base rates, underreporting, and/or inaccurate reporting. Measuring FE risk is one way to overcome some of these inherent challenges, yet few measures exist that do so. One measure that holds great promise is the Financial Exploitation Vulnerability Scale, and its short-form (FEVS, FEVS-SF, respectively). The present study examined the measurement property evidence for a Hebrew version of the FEVS and FEVS-SF.

**Research Design and Methods:**

Participants (*N *= 1281; *M* age = 69.98; *SD *= 7.39) from four separate studies were included in an amalgamated sample. Participants completed basic demographic measures, the FEVS in Hebrew (from which the FEVS-SF was derived), and a questionnaire on self-reported history of FE. Factor structure (structural validity), internal consistency estimates, and discriminative (known groups) construct validity of the FEVS and FEVS-SF were examined.

**Results:**

A clear factor structure was not found for the FEVS, but a two-factor solution yielded a good model fit for the FEVS-SF, with two conceptually meaningful factors identified (financial well-being and financial decision making). Reliability estimates were acceptable for both the FEVS (0.77) and FEVS-SF (0.81). Discriminative validity was also supported; participants with a self-reported history of FE scored significantly higher on both measure versions compared to participants denying a history of FE.

**Discussion and Implications:**

Findings support the use of the FEVS-SF in Hebrew to measure FE vulnerability. The measure can be administered easily and at low cost, and may serve useful to researchers and clinicians alike.

## Background and objectives

Financial exploitation (FE), or the unlawful taking or inappropriate use of a person’s funds, properties, or assets, is of particular concern for older adults (Wood & Lichtenberg, 2017). FE results in devastating consequences for victims of FE, including victims who are older adults, as well as their close others (e.g., families, caregivers) and society at large (Spreng et al., 2016). From a financial perspective, based on estimates from the United States, older adults are approximated to lose up to 28.3 billion dollars annually to FE (Gunther, 2023). Significant mental health (Lavery et al., 2021), physical health (Burnett et al., 2016), and social and community consequences (Nguyen et al., 2021) have been documented. As such, FE assessment tools are of critical importance both for advancing research of the phenomenon as well as developing effective prevention and intervention strategies.

Assessing FE in older adults is challenging for several reasons. First, underreporting of FE has been widely documented in the literature (Acierno et al., 2020; Jackson & Hafemeister, 2013; Lachs & Berman, 2011). This can occur for various reasons including a desire to hide the FE occurrence due to embarrassment and/or shame, a lack of awareness that the FE experience occurred, or fear of retaliation by the offender (i.e., if perpetrated by a close other; Jackson & Hafemeister, 2013; Lachs & Berman, 2011). Such factors can impact the effectiveness of both research and clinical work, as individuals may underreport or incorrectly report experiences of FE. An additional challenge in assessing FE, particularly in research settings, relates to the relatively lower base rate of FE. Prevalence studies suggest that approximately 1 in 20 older adults will experience FE per year (Burnes et al., 2017; Pillemer et al., 2016). While this is, at face value, an alarmingly high number when considering the rapidly growing older adult population, a 5% base rate leads to challenges in recruiting sufficiently powered samples of older adults for studies. For example, based on this number, in a sample of 300 older adults, only 15 older adults are likely to report an experience of FE. Such issues are further compounded by longitudinal investigations, as change in FE status over time will be minimal even in very large samples, and thus may not provide the power needed to examine FE correlates longitudinally.

To overcome some of these challenges, researchers have sought alternative approaches to assessing FE, including the development of assessment tools that measure risk of FE. The Financial Exploitation Vulnerability Scale (FEVS), by Lichtenberg et al. (2020), is one such tool which may provide a viable solution to some of the issues inherent in assessing FE in older adults. The self-report, self-administered measure includes 17 items which focus on a person’s contextual financial reality and provides a total risk score which reflects the sum of responses to the 17 items. Following publication of the 17-item version, a 9-item short-form version was introduced by Campbell & Lichtenberg (2021).

There are many benefits of a FE vulnerability measure with supporting measurement property evidence (e.g., reliability, validity). First, this type of measure overcomes the issue of underreporting as individuals are not asked to directly report on an experience of FE but are instead asked to respond to questions assessing various contextual risk factors of FE. Second, all participants receive a risk score, thereby increasing the statistical power available to investigate FE. When considering longitudinal examinations of FE overtime, such a measure may prove especially useful, assuming evidence of test-retest reliability and longitudinal validity, given that even subtle changes in risk level may be predicted by or predictive of other psychosocial, cognitive, and behavioral factors. Finally, from a clinical perspective, such a measure may also assist clinicians in identifying individuals at high-risk of experiencing FE who may benefit from targeted interventions, ideally even before an FE experience occurs.

Despite the potential benefits of a FE risk measure for both clinical and research purposes, current evidence for the FEVS has been limited to studies within American older adult samples. Hence, the aim of this study was to examine the measurement property evidence for a Hebrew version of the FEVS in a sample of older adults in Israel. The objectives were to examine: (1) the factor structures (i.e., structural validity) of the Hebrew version of the FEVS and short-form FEVS; (2) the internal consistency of the FEVS and short-form FEVS (i.e., internal reliability); and (3) whether the scores of the FEV and FEVS-SF correlate with groups who self-report having experienced FE or not (i.e., hypothesis testing for known groups/discriminative construct validity). To this end, four separate datasets collected over a 3-year period were harmonized and merged to produce a large sample of adults living in Israel, aged 60 and over who responded to the Hebrew version of the FEVS, and who self-reported their history of FE experiences. The goal of these analyses was to examine the possible supporting measurement property evidence of the Hebrew version to be used for descriptive measurement purposes, as measurement property evidence cannot be assumed stable across different measurement instrument language versions (de Vet et al., 2011).

A priori hypotheses, including the direction and magnitude of relationships where applicable as guided by current measurement property evidence methods standards (de Vet et al., 2011), were as follows:

- Objective 1: (H1) Since past evidence does not support a one-factor solution of the FEVS (Campbell & Lichtenberg, 2021), we predicted that a multiple factor solution may be ideal. (H2) We further hypothesized a single factor solution for the short form given how it was derived (Campbell & Lichtenberg, 2021).
- Objective 2: (H3) We hypothesized that the internal consistency estimates would be similar to those reported in the USA studies that used the English version, as there were no theoretical reasons why the language or cultural differences would influence the inter-relatedness of a FE measure. (H4) We hypothesized that for each individual study, each having a unique sample, and across the harmonized sample, internal consistency estimates would be similar.
- Objective 3: (H5) We hypothesized a moderate relationship (i.e., Cohen’s *d* effect sizes ranging from 0.50 to 0.79; [Cohen, 2013]) with reported history of FE as this measure asks about actual experiences of FE and, therefore, may relate to risk but does not fully overlap with the construct.

While there are several published studies that have utilized the Hebrew version of the FEVS to examine risk factors of FE (e.g., Weissberger & Bergman, 2024; Weissberger et al., 2023), this is the first study to formally consider the internal consistency and factor structure of the FEVS in Hebrew, and examine its construct convergent validity with another commonly utilized tool for assessing FE (self-reported history of FE; e.g., Weissberger, 2022; Weissberger et al., 2020). If reliability estimates prove strong, findings from this study will reinforce the use of this tool in both research investigations and clinical practices within Israel, and may encourage other international researchers to translate and adopt the assessment in their own countries.

## Research design and methods

This study aligns with the measurement property (i.e., psychometric) evidence taxonomy and study design checklist from the COnsensus-based Standards for the selection of health Measurement Instruments (COSMIN), the internationally leading group guiding measurement science (Mokkink et al., 2019). Within this guidance, the study was analyzed within classical test theory (CTT) approaches (de Vet et al., 2011; Mokkink et al., 2019), which align with the measurement development and past measurement property evidence studies examining the English versions of the FEVS and the FEVS-SF (Campbell & Lichtenberg, 2021; Lichtenberg et al., 2020).

### Participants and procedures

A total of 1,281 participants (*M* age = 69.98; *SD *= 7.39; range = 59–101; 67.80% female) from across four separate studies were included in the present amalgamated sample for this study. The four studies were aimed at understanding psychosocial risk factors of FE and FE vulnerability and have resulted in a number of publications (e.g., Weissberger & Bergman, 2022, 2024, 2025; Weissberger et al., 2023). Data collection periods were as follows: Study 1, 2021–2022; Study 2, 2022–2023; Study 3, 2023–2024; Study 4, 2023–2024. For all studies, convenience-sampling methods were used such that student research assistants approached eligible community-living participants residing in Israel. Inclusion and exclusion criteria were similar for all four studies. Specifically, participants had to be 60 years old or older (though three participants were age 59 across the four samples) and self-report to speak and read Hebrew fluently. Across all studies, participants were excluded if they had a known or diagnosed cognitive or neurological disorder or a major psychiatric illness (e.g., requiring treatment or hospitalization), as the studies focused on healthy older adults without cognitive impairment. Participants recruited into the studies were provided with a designated Qualtrics link which led them to an online consent form. After providing consent, participants were led to the online questionnaires. All studies received ethical approval from the Institutional Review Board of Bar-Ilan University.

### Measures

To merge the datasets, demographic variables common across all four studies were first identified. For certain variables (e.g., income), minor coding differences between datasets were resolved by recategorizing collected continuous data into response options consistent between datasets. Participants (*n *= 85) who were missing data on the two main variables of interest, the FEVS or self-reported history of FE, were excluded from the combined dataset. This resulted in a final sample of 1,281 participants. The measures included are as follows:

#### Financial exploitation vulnerability scale

The FEVS was developed by Lichtenberg et al. (2020) as a measure which captures a person’s degree of vulnerability to experiencing FE. As stated by Lichtenberg et al., the scale focuses on “a person’s perception of their financial security and psychological and relationship insecurity in relation to personal finance” (p. 2). The measure was empirically derived from the 68-item Lichtenberg Financial Decision Rating Scale (LFDRS) within a CTT measurement model (de Vet et al., 2011). From 34 contextual items identified in the LFDRS, the 17-item measure (the FEVS) was derived by selecting items which statistically differentiated FE victims from nonvictims. The developers of the FEVS reported acceptable measurement property (i.e., psychometric) evidence on a sample of older adults over the age of 60 living in the United States, of which 78 self-reported FE and 164 did not report a history of FE. Forty of the 78 FE cases were verified by reviewing bank statements and credit card reports, and the other cases were verified via follow-up questions to assess validity of the FE experiences. Internal consistency (Cronbach’s alpha = 0.82) indicated an adequate degree of inter-relatedness of the items. Criterion validity was also reported via Receiver Operating Curve analysis. The analysis revealed adequate sensitivity and specificity for identifying FE victimization (AUC = 0.814, 95% CI [0.757–0.871]). In a cross-validation study (Moray & Lichtenberg, 2025) which included a sample of 114 participants from the Detroit community or recruited from a financial coaching service (SAFE; see Moray & Lichtenberg, 2025 for details), the measure also demonstrated adequate internal consistency (Cronbach’s alpha = 0.80), and acceptable sensitivity and specificity for detecting FE (AUC = 0.68, 95% CI: 0.57–0.79). In another study (Campbell & Lichtenberg, 2021) which conducted an analysis of factor structure, fit indices were found to fall in the poor range (RMSEA = 0.10; TLI = 0.87).

For this present study, the study first author GHW and another independent fluent Hebrew-English researcher from Israel translated and back-translated this measure for use in Hebrew and have published several studies utilizing the measure (e.g., Weissberger & Bergman, 2024; 2025; Weissberger et al., 2023). Both translators independently translated the measure, and disagreements in the translation were resolved during a consensus meeting in which the disagreements were discussed until a consensus decision could be made. While the FEVS can be administered in an interview format, participants in all four studies self-completed the questionnaire in the format of an online survey which included other self-report questionnaires. For this measure, participants are asked to respond to 17 items with varying response options (e.g., “not at all worried,” “somewhat worried,” “very worried”; or “satisfied,” “neither satisfied or dissatisfied,” or “dissatisfied”). For example, participants are asked “Has a relationship with a family member or friend become strained due to finances as you have gotten older?” and “How likely is it that anyone now wants to take or use your money without your permission?” Scores on this measure are summed, with higher scores indicating higher FE vulnerability. For a full list of items in Hebrew, see Supplementary Appendix A; for original items in English please see Lichtenberg et al. (2020).

All datasets utilized the FEVS. Thus, to specifically examine study objectives with the FEVS short form (FEVS-SF), we selected out the nine items identified by Campbell and Lichtenberg (2021) as part of the short form and tested hypotheses with just these items included. As with the full version, scores on the 9-item FEVS-SF are summed to indicate one’s degree of FE vulnerability. It is important to note that the FEVS-SF requires the administration of 10 items because one item (“how satisfied are you with this money management arrangement”) refers to a question that is not included in the final summed score (“who manages your money?”).

The short-form was derived by an exploratory factor analysis with direct oblimin rotation conducted on the full 17-item version (see Campbell and Lichtenberg, 2021). Items with factor loadings of 0.3 or greater were retained. This resulted in a 9-item scale explaining 45.7% of the variance. Internal consistency was 0.82 and like the full version, the scale was shown to have good ability to differentiate 78 FE victims (verified via bank and credit card statements) from 164 non-FE victims (AUC = 0.79; 95% CI: 0.72–0.85; Campbell & Lichtenberg, 2021).

### Self-reported history of FE

To divide participants into dichotomous groups who had or had not experienced FE (i.e., for known groups/discriminative analysis), participants were asked two questions to assess their history of FE (Weissberger, 2022; Weissberger et al., 2020): (1) “After the age of 50, was there a situation in which you felt you were taken advantage of financially?”; (2) “After the age of 50, did someone you know feel that you were taken advantage of financially?” Participants who provided an affirmative response to either question were included in the FE group. Age 50 was selected as a cut-off given research suggesting that pre-retirement and middle age are particularly vulnerable times for individuals in the context of FE (Beach et al., 2023; DeLiema, 2024; Mueller et al., 2020; Nolte et al., 2021).

#### Other variables of interest

Age (in years) and sex (male = 0; female = 1) were assessed for all participants. Participants were asked to indicate their education level (1 = completed grade school; 2 = partial high school; 3 = completed high school; 4 = partial undergraduate studies; 5 = bachelor’s degree; 6 = partial graduate school; 7 = master’s degree; 8 = PhD) and their gross combined monthly household income. Response options ranged from 0 (no income) to 11 (over 30,001 New Israeli Shekels [NIS]). Finally, participants were asked to review a list of 14 medical diagnoses and indicate which of them they have. The sum of the illnesses endorsed was used in analyses.

### Statistical analyses

All statistical analyses were conducted in R Statistical Software (v4.4.2; R Core Team, 2024). Descriptive statistics for all variables of interest were first calculated. Then, to address the first objective (H1, H2), confirmatory factor analyses for the full FEVS and the short-form were conducted using the lavaan package in R. The models were estimated using robust maximum likelihood estimation. Poor model fit led us to conduct exploratory factor analyses using the psych package in R. We used oblimin rotation and iteratively constrained the model to 2, 3, or 4 factors to identify the best model fits as we assumed that factors may be interrelated. For factor extraction, factors with eigenvalues of 1 or higher were retained. A scree plot was also examined to look for change in the slope of the eigenvalues. Finally, specific items that loaded strongly onto the factors identified were examined to see if the items fit conceptually together.

To test H3 and H4 of Objective 2, internal consistency for the FEVS and FEVS-SF across the four datasets and the amalgamated sample was calculated. To this end, Cronbach’s alpha was used to examine internal consistency in the full scale (FEVS and short form) as well as any factors (subscales) identified in the factor analyses.

Finally, to test H5 of Objective 3, independent samples *t*-tests comparing participants with a self-reported history of FE to those without a self-reported history of FE on both the long and short form versions of the FEVS were conducted.

Reporting was guided by the COSMIN reporting standards for measurement property evidence studies (Gagnier et al., 2021).

## Results

### Participant characteristics and bivariate analyses of study variables

Participant characteristics for the pooled sample and separately for the four individual datasets are presented in Table 1. In the pooled sample (*N *= 1,283), participants reported, on average, having completed or partially completed a post-secondary Bachelor’s degree; this was consistent with the mode of the sample. Participants reported an average gross combined monthly household income of about 10,000 NIS per month (slightly below the average monthly income; Israel Central Bureau of Statistics, cbs.gov.il), though the mode in the sample was higher (17,001–24,000). They also reported about two diagnosed medical illnesses. The sample was predominantly female (67.8%), and 20.4% of the sample reported a past perceived experience of FE.

**Table 1:** Participant characteristics and scores on the FEVS and FEVS-SF for the pooled sample (*N *= 1283) and each separate dataset.

Dataset	Data collection year	Sample size (% FE)	Age	Sex	Education	Income	Sum of illnesses	FE	FEVS	FEVS-SF
			*M* (*SD*; range)	% female	*M* (*SD*; range)	*M* (*SD*; range)	*M* (*SD*; range)	% yes	*M* (*SD*; range)	*M* (*SD*; range)
**Pooled**	—	1283 (20.4%)	69.98 (7.39; 59–101)	67.80%	4.43 (1.93; 1–8)	6.98 (2.73; 0–11)	2.08 (1.55; 0–12)	20.4%	6.13 (4.07; 0–22)	4.50 (3.22; 0–18)
**1**	2021–2022	128 (21.9%)	67.18 (5.69; 60–94)	58.46%	4.53(1.92; 1–8)	7.44 (2.63; 0–11)	2.02 (1.83; 0–12)	21.9%	5.56 (3.99; 0–21)	4.24 (3.25; 0–18)
**2**	2022–2023	152 (19.1%)	66.99 (6.47; 60–92)	60.40%	4.28 (1.93; 1–8)	7.14 (2.42; 0–11)	1.84 (1.47; 0–6)	19.1%	6.38 (4.34; 0–22)	4.81 (3.53; 0–17)
**3**	2023–2024	631 (19.0%)	72.52 (7.71; 59–101)	69.02%	4.31 (1.96; 1–8)	6.66 (2.76; 0–11)	2.16 (1.36; 1–8)	19.0%	6.14 (4.17; 0–22)	4.50 (3.23; 0–17)
**4**	2023–2024	370 (22.7%)	67.85 (6.17; 60–86)	72.63%	4.66 (1.86; 1–8)	7.29 (2.78; 0–11)	2.08 (1.72; 0–9)	22.7%	6.21 (3.79; 0–22)	4.47 (3.06; 0–16)

*Note*. FE = financial exploitation; FEVS = Financial Exploitation Vulnerability Scale; FEVS-SF = Financial Exploitation Vulnerability Scale-Short Form; *M* = mean; *SD* = standard deviation.

With regard to bivariate associations between study variables, in the pooled sample, higher scores on the FEVS and FEVS-SF were associated with lower education (*r *= −0.23, *p* < .001; *r *= −0.21, *p* < .001, respectively) and lower income (*r *= −0.34, *p* < .001; *r *= −0.34, *p* < .001, respectively). The FEVS and FEVS-SF were not associated with age (*p* = .722 and *p* = .164, respectively). Women scored marginally higher than men on the FEVS [*t*(1085) = −1.75, *p* = .081] and significantly higher than men on the FEVS-SF [*t*(1085) = −2.90, *p* = .004]. Higher scores on the FEVS and FEVS-SF were also associated with a significantly higher number of illnesses (*r *= 0.20, *p* < .001; *r *= 0.17, *p* < .001, respectively). For correlations with demographic variables for each dataset separately, please see Supplementary Table 1, see online supplementary material.

### Factor structure of the FEVS and FEVS-SF

A confirmatory factor analysis of the FEVS was conducted to examine the fit of a one-factor model. Model fit statistics indicated poor fit to the data: Robust χ^2^_119_ = 863.67 with a CFI = 0.79, TLI = 0.76, RMSEA = 0.08, and a SRMR = 0.06. Standardized estimates of factor loadings indicated variability in associations to the latent factor. While some items loaded strongly onto the latent factor (e.g., FEVS item 1 = 0.71, item 13 = 0.74) others loaded weakly (e.g., FEVS item 3 = 0.083; FEVS item 7 = 0.112). Supplementary Table 2a (see online supplementary material) displays these standardized factor loadings.

The CFA of the FEVS-SF revealed a slightly stronger model fit, with Robust χ^2^_27_ = 273.86 CFI = 0.91, TLI = 0.88, RMSEA = 0.09, and a SRMR = 0.05. Variability of standardized factor loadings was less wide than the full version, ranging between 0.51 and 0.72, with the exception of one item (Item 10) which had a weaker loading of 0.22. Supplementary Table S2b (see online supplementary material) displays these standardized factor loadings.

Due to the relatively poor fit observed for each measure using a one-factor solution CFA, we next conducted exploratory factor analyses for two-, three-, and four-factor solutions for the FEVS and FEVS-SF. Model fit statistics are summarized in Table 2.

**Table 2:** Model fit statistics of exploratory factor analyses for the 2-, 3-, and 4-factor solutions of the FEVS and FEVS-SF.

	FEVS			FEVS-SF		
	2-factor	3-factor	4-factor	2-factor	3-factor	4-factor
**Chi-square**	638.58	314.83	214.40	105.54	39.26	10.98
**BIC**	−98.59	−314.98	−314.21	−30.44	−46.62	−31.97
**TLI**	0.83	0.92	0.94	0.95	0.97	0.99
**RMSEA**	0.06	0.05	0.04	0.06	0.04	0.03

*Note*. BIC = Bayesian Information Criterion; FEVS = Financial Exploitation Vulnerability Scale; RMSEA = Root Mean Square Error of Approximation; TLI = Tucker-Lewis Index.

For the FEVS, model fit of the two-factor solution was poor and improved slightly for the three-factor solution, and was best with the four-factor solution (χ^2^_103_ = 215.40, BIC = −98.59, TLI = 0.94, RMSEA = 0.04). Examination of the scree plot and corresponding eigenvalues showed a steep drop from the first eigenvalue (3.71) to the second eigenvalue (0.90). Next, we examined factor loadings of the four-factor solution to determine whether the items fit into clear conceptual factors. Factors three and four accounted for only 4.4% and 2.2% of variance in the observed items, and factor loadings were low (<0.15) for many of the items on each of these factors. A similar picture arose for the three-factor solution. For the two-factor solution, the second factor only accounted for 5.1% of variance and there were items that did not load strongly onto any factor with factors loadings (e.g., item 3, loading of 0.12). Given these results, it was determined that certain items may need to be removed in order to reach an optimal factor solution that is conceptually meaningful and structurally sound. Factor loadings for the 2-, 3-, and 4-factor solutions can be viewed in Supplementary Tables 3–5, see online supplementary material.

With regards to the FEVS-SF, the two-factor solution had an adequate fit with the best fit seen in the four-factor solution (χ^2^_6_ = 10.98, BIC = −31.97, TLI = 0.99, RMSEA = 0.03). As with the long form, the FEVS-SF also showed a steep drop in eigenvalues from the first factor (3.23) to the second (0.47). In order to determine the best factor solution, factor loadings were examined. In the four-factor solution, the factors accounted for 55.5% total variance. However, factors three and four had very low factor loadings (lower than 0.20) and factor four accounted for only 4% of the variance in the observed items. Thus, we determined that while the fit indices were best for the four-factor solution, this was not the optimal fit for the FEVS-SF. Analogous results were found for the three-factor solution which accounted for 43.6% of variance in observed items. Specifically, factor 3 accounted for only 11.7% of total variance and yielded low factor loadings (below 0.20). For the two-factor solution, 37.3% of total variance was accounted for by the two factors, with each contributing relatively equally to this total (Factor 1, 19.9% and Factor 2, 17.4%). Based on examination of the items, it was determined that the two-factor solution was the best solution for the FEVS-SF. Factor loadings for each item can be viewed in Table 3.

**Table 3:** Factor loadings for each item of the 2-factor solution of the FEVS-SF. Items that loaded more strongly onto factor 1 are highlighted in grey.

Item	Question	Factor 1	Factor 2
**FEVS 1**	How worried are you about having enough money to pay for things?	0.683	
**FEVS 2**	Overall, how satisfied are you with your finances?	0.799	
**FEVS 4**	How satisfied are you with this (money management) arrangement?	0.210	0.363
**FEVS 5**	How confident are you in making big financial decisions?		0.570
**FEVS 6**	How often do you worry about financial decisions you’ve recently made?	0.187	0.549
**FEVS 8**	How often do your monthly expenses exceed your regular monthly income?	0.532	
**FEVS 10**	How often do you wish you had someone to talk to about financial decisions, transactions, or plans?	−0.204	0.468
**FEVS 11**	How often do you feel anxious about your financial decisions and/or transactions?		0.704
**FEVS 13**	How often do you feel downhearted or blue about your financial situation or decisions?	0.518	0.285

*Note*. FEVS = Financial Exploitation Vulnerability Scale. In reviewing the items constructs to factor loadings, theoretically it seems Factor 1 relates to a conceptualization of financial well-being and Factor 2 related to conceptualizations of feelings about financial capability (i.e., knowledge, skills, decision-making, or behaviors).

In an additional set of follow-up exploratory analyses, we examined measurement invariance of the FEVS-SF for sex, income, and education using multi-group confirmatory factor analysis with the Weighted Least Squares Mean and Variance (WLSMV) estimator (Hirschfeld & Von Brachel, 2014). Of note, Maximum Likelihood Robust was used for primary structural models given the large sample size, whereas WLSMV was used for invariance testing to appropriately model item thresholds given the 0–2 ordinal response format of the items. Income and education were divided into high vs. low groups using a median split of the data. For each variable, invariance was tested sequentially by examining change in CFI between the configural model (baseline model) and the metric model (equal loadings), between the metric model and the scalar model (equal loadings and thresholds), and between the scalar model and the strict model (equal loadings, thresholds, and residuals). Results are displayed in Supplementary Table 6 (see online supplementary material). Findings support full measurement invariance across sex, education, and income as change in CFI and RMSEA values were all below recommended cutoffs of 0.01 and 0.015, respectively (see Chen, 2007; Cheung & Rensvold, 2002).

### Internal consistency of the FEVS and FEVS-SF

Estimates of internal consistency were calculated for the FEVS and FEVS-SF using the pooled sample. This revealed adequate internal consistency for both forms of the questionnaire. Specifically, for the FEVS, Cronbach’s alpha was 0.77 [95% CI: 0.75, 0.79]. For the FEVS-SF, Cronbach’s alpha was 0.81 [95% CI: 0.80, 0.83].

Internal consistency was also calculated for each dataset separately. For the FEVS, Cronbach’s alpha values ranged from 0.73 to 0.80. Slightly higher reliability estimates were found for the FEVS-SF with Cronbach alpha values ranging from 0.79 to 0.86 across the four datasets. Table 4 displays internal reliability estimates of the FEVS and FEVS-SF separately for each of the four datasets.

**Table 4:** Reliability estimates of the FEVS separately for each of the four datasets used in the present study.

Dataset	FEVS		FEVS-SF	
	Cronbach’s alpha	Feldt 95% CI	Cronbach’s alpha	Feldt 95% CI
**Pooled**	0.77	0.75–0.79	0.81	0.80–0.83
**1**	0.77	0.72–0.83	0.82	0.77–0.86
**2**	0.80	0.76–0.85	0.86	0.82–0.89
**3**	0.79	0.77–0.81	0.81	0.79–0.83
**4**	0.73	0.69–0.76	0.79	0.75–0.82

*Note*. CI = confidence interval; FEVS = Financial Exploitation Vulnerability Scale.

Given a strong two-factor solution of the FEVS-SF, showing adequate factor loadings for theoretical conceptualizations of financial well-being (factor 1) and financial decision-making/behavior (factor 2), we examined internal reliability estimates separately for these two factors in the pooled sample. The well-being factor (i.e., factor 1) demonstrated adequate internal consistency (0.78, 95% CI [0.76, 0.80]). Internal consistency for factor 2 (decision making/behavior subscale; items 4, 5, 6, 10, and 11) was lower, 0.68 (95% CI [0.65, 0.71]).

### Discriminative construct validity with perceived history of FE

An independent samples *t*-test revealed that the FE group had significantly higher FEVS scores than the non-FE group (*p* < .001) in the amalgamated sample. This was also the case for the FEVS-SF (*p* < .001). Each separate dataset showed significant FE versus non-FE differences in FEVS scores (all *p*s ≤ .001) with the exception of dataset 2 (*p* = .317). As was the case with the FEVS, group differences between FE and non-FE participants were found on the FEVS-SF for all datasets (all *p*s ≤ .002) with the exception of dataset 2 (*p* = .280). Results of the *t*-tests for the pooled sample and each separate dataset are summarized in Table 5.

**Table 5:** Results of *t*-tests comparing FE to non-FE participants on the FEVS and FEVS-SF for the pooled sample and each dataset separately.

Dataset	Measure	FE vs non-FE		
		*t* statistic	*p*-value	Cohen’s *d*
**Pooled**	FEVS	−10.57	<.001	0.733
	FEVS-SF	−8.53	<.001	0.592
**1**	FEVS	−3.261	.001	0.700
	FEVS-SF	−3.191	.002	0.682
**2**	FEVS	−1.005	.317	0.210
	FEVS-SF	−1.084	.280	0.224
**3**	FEVS	−8.721	<.001	0.890
	FEVS-SF	−6.143	<.001	0.623
**4**	FEVS	−6.065	<.001	0.750
	FEVS-SF	−5.526	<.001	0.686

*Note*. FE = financial exploitation; FEVS = Financial Exploitation Vulnerability Scale; FEVS-SF = Financial Exploitation Vulnerability Scale-Short form.

We also examined differences between FE and non-FE groups for the two FEVS-SF factors identified from the EFA. For the decision making/behavior factor, the FE group scored higher (*M *= 2.45; *SD *= 1.88) than the non-FE group (*M *= 3.31; *SD *= 1.76) a statistically significant difference [*t*(1279) = −6.95, *p* ≤ .001; Cohen’s *d* = 0.48]. The same was seen for the well-being factor, with the FE group scoring significantly higher (*M *= 1.67; *SD *= 1.94) than the non-FE group [*M *= 2.67; *SD *= 1.75; *t*(1279) = −8.03, *p* ≤ .001; Cohen’s *d* = 0.56]. To summarize, while we hypothesized a moderate relationship (i.e., Cohen’s *d* effect sizes ranging from 0.50 to 0.79) comparing the two groups of those reporting FE history and those not, often this hypothesis was supported but there were instances where the discriminative validity (known groups) was lower than what was hypothesized.

### Additional exploratory and sensitivity analyses

As an exploratory investigation of convergent validity, we examined whether the two factors diverged in their association with income. Theoretically, we expected the well-being factor to be more strongly related to income than the decision-making factor. In order to explore this possibility, we ran two linear regression models, one for financial well-being and one for decision making, with income as the predictor variable. For both factors, higher income was associated with lower scores. Importantly, the association was stronger for the FEVS-SF well-being factor (*R*^2^ = 0.16, *b *= −0.27, *β* = −0.39, *p *< .001) than for the decision making/behavior factor (*R*^2^ = 0.04, *b *= −0.21, *β* = −0.27, *p *< .001).

Given potential heterogeneity between the four datasets, we conducted sensitivity analyses to examine whether this may have affected the overall construct validity findings for the FEVS and FEVS-SF. To this end, we reran the analyses using multiple regression models which included dataset (treated as a categorical variable) and the interaction between dataset and FE group. For the FEVS, comparison of the models with and without an interaction term was statistically significant [*F*(3, 1273) = 2.86, *p *= .036], indicating some variation in the magnitude of the FE group effect across datasets; however, the increase in explained variance was small (Δ*R*^2^ = 0.006), the interactions themselves were non-significant, and the direction of the FE group difference was consistent across datasets. For the FEVS-SF, addition of the FE group by dataset interaction effect to the regression model did not significantly explain additional variance in FEVS-SF scores, *F*(3, 1273) = 1.06, *p* = .36. These findings suggest that the construct validity results are largely robust across samples.

## Discussion and implications

This study examined the factor structure (structural validity), internal consistency (internal reliability), and discriminative (known groups) construct validity (hypothesis testing) of the Hebrew translated versions of the FEVS (Lichtenberg et al., 2020) and short form version of the FEVS (FEVS-SF; Campbell & Lichtenberg, 2021) in a pooled sample of community living Israeli older adults who participated in one of four studies over the course of four years. Consistent with our first hypothesis, confirmatory factor analysis showed that a single factor structure was not an ideal fit for the FEVS. Exploratory factor analysis demonstrated that 2-, 3-, and 4-factor solutions were also suboptimal. In contrast to our second hypothesis that the FEVS-SF would load onto a single factor, we found that a two-factor solution yielded the best model fit, with two conceptually meaningful factors identified. With regards to our third and fourth hypotheses regarding internal consistency, reliability estimates were acceptable for both the FEVS and the FEVS-SF, as well as each subfactor of the FEVS-SF identified by the exploratory factor analysis. Finally, consistent with our fifth hypothesis, statistically significant *t* tests revealed discriminative validity between self-reported history of FE and the FEVS as well as FEVS-SF and its empirically identified subscales.

Results of the factor analyses suggest that certain items in the full version FEVS in Hebrew may need to be removed in order to reach an adequate model fit with factors that are conceptually meaningful and explain significant variance in the measure. This finding is not inconsistent with Campbell & Lichtenberg (2021), who derived a short-form FEVS after conducting a factor analysis on the longer version which revealed weak factor loadings for certain items of the 17-item measure. Encouragingly, the short-form FEVS in Hebrew evidenced adequate model fit in a two-factor solution.

Based on the specific questions that loaded onto each factor and review of the theoretical or conceptual ideas of items within each factor, we identified two current evidence-based and internationally recognized financial models or frameworks with which the two factors seem to align. First, items of Factor 1 aligned well with assessing a concept of financial well-being which describes an experience wherein one is able to meet their own and their dependents’ current and reasonably anticipated future financial obligations; anticipate a level of security in meeting their future financial demands and shocks, should they happen; experience a level of choice or control in their financial lives and decisions; and find some level of life satisfaction or quality of life from their financial situations (Consumer Financial Protection Bureau/USA [CFPB], n.d.; Kempson & Poppe, 2018). Thus, financial wellbeing can be both an objective experience (e.g., high asset to debt ratios) or subjective experience (e.g., feeling financially well and able to meet financial demands), with all people experience differing and changing levels of financial wellbeing. The items of factor 1 seemed to align with subjective perceptions of one’s own level of financial wellbeing.

The items of Factor 2 seemed to align with assessing a concept of financial capability, which is inclusive of financial literacy (knowledge), skills, decision-making, attitudes, and applied or real-world behaviors that are completed within a specific context and within social structures for each individual or collective (Kempson & Poppe, 2018; Kempson et al., 2006; Xiao et al., 2022). Thus, while the two concepts are intertwined, they are also unique in that capability is the knowledge, decisions, and behaviors of an individual in context, and financial wellbeing is the outcome of financial capability and other factors within a specific context (Kempson & Poppe, 2018). This is interesting as the FEVS and FEVS-SF, as measures of FE risk, may be delineating an inherent relationship of financial capability and financial well-being to FE, where less capability and lower well-being place individuals at increased risk for FE.

The finding of two factors in the FEVS-SF, financial wellbeing and financial capability/decision-making, as being related to financial vulnerability is interesting to ponder conceptually. Financial vulnerability in other financial literature has been defined as an individual’s or collective’s (e.g., family unit, community, geo-political area) experience or risk of financial distress (Buckland & Visano, 2022). It is therefore reasonable to expect that these two factors would demonstrate relationships to a scale of FE risk or vulnerability. However, the added relationship to FE highlights the unique but inter-relatedness of financial vulnerability (inclusive of financial wellbeing and capability/decision-making) with FE. Thus, while the statistical analyses demonstrated relationships, our findings also suggest there is some uniqueness to these concepts, with other factors most likely also being implicated in the total relationship between financial capability (decision making/behavior), financial well-being, and FE risk or experience. In other literature, both individual factors, such as personality traits, attitudes, and cognition, as well as environment or context factors, such as access to financial social supports and reliable information, exposure to financial threats, and stable economic resources, have been implicated as other factors influencing these inter-related concepts (Buckland & Visano, 2022; Kempson & Poppe, 2018; Xiao et al., 2022).

The internal reliability values found in this study align well with those reported in the literature for the FEVS and FEVS-SF. In their manuscript introducing the measure to the scientific community, Lichtenberg et al. (2020) reported good internal consistency (Cronbach’s alpha = 0.82). While our value for the full combined sample was slightly lower (Cronbach’s alpha = 0.77), this value is considered adequate according to well-accepted standards regarding internal reliability (Tavakol & Dennick, 2011). Similarly, the FEVS-SF demonstrated to have good internal consistency by Campbell and Lichtenberg (2021); Cronbach’s alpha = 0.85, as was found in this study (Cronbach’s alpha = 0.81), and the financial well-being and financial decision-making factors also evidenced adequate reliability (0.78 and 0.68, respectively). Thus, it can be concluded that both long and short forms of the measure demonstrate acceptable internal consistency for use in Hebrew. In considering both factor analysis results and reliability estimates, the two-factor solution of the FEVS-SF may be an optimal measure for use in Hebrew as it evidenced a good overall fit and strong internal consistency.

Our third study objective was to examine whether the FEVS and FEVS-SF can discriminate between groups of people who have or have not experienced FE, as determined by another commonly utilized method of assessing FE in the research literature of self-reported history of FE experiences (e.g., Weissberger, 2022; Weissberger et al., 2020). Across multiple datasets and the pooled sample, individuals with a self-reported history of FE scored significantly higher on both versions of the FEVS. Consistent with our prediction that effect sizes would be moderate in size, effect sizes in this study were mostly moderate in size, supporting a robust relationship between self-reported FE status and FEVS/FEVS-SF; exceptions to this were dataset two which did not yield significant differences and dataset 3 which yielded a large effect size of 0.89 (Cohen, 2013). Subfactors of the FEVS-SF (decision making; well-being) also revealed moderate discriminative validity, with the well-being factor showing a slightly stronger effect. Future studies may consider examining the ability of each of these factors to differentially predict FE.

Overall, these results align with the method by which the FEVS was constructed. Specifically, items of the FEVS were selected if they statistically differentiated FE victims from nonvictims (Lichtenberg et al., 2020). However, it is important to note that assessing self-reported FE is not without its own limitations, and the noise introduced by this assessment technique may have at least partially contributed to the wide range in effect sizes and lack of an effect in dataset two. For example, self-reported FE may be influenced by response biases that arise for various reasons including shame and embarrassment in admitting to an experience of FE, and fear of retaliation by perpetrators (Jackson & Hafemeister, 2013; Lachs & Berman, 2011). Additionally, self-report relies on an individual’s own knowledge of what constitutes a FE experience, and may therefore be prone to over- or under-reporting. Thus, while these findings support the discriminative validity of the FEVS and FEVS-SF, it will be important to also establish their criterion validity. For example, examining the ability of the measures to predict actual confirmed FE experiences will further validate its use in measuring FE vulnerability as determined by a more robust or “gold-standard” measure of FE. It is also important to note that FE vulnerability does not necessarily lead to actual experienced FE, but may predict other important outcomes such as development of cognitive impairment and/or Alzheimer’s disease (Fenton et al., 2022). Thus, further validating the FEVS and FEVS-SF will be a critical aim of future studies as doing so will enhance the utility of the measures in clinical settings in which identifying true victims of FE is a key part of providing treatment and intervention to those most in need.

Analyses confirmed that the FEVS-SF has a sound two-factor structure, good reliability for use in Hebrew, and that it demonstrates convergent validity with the self-reported history of FE measure. In addition, supplementary analyses examining measurement invariance also support full measurement invariance for sex, income, and education; future studies should confirm cross-cultural validity for the FEVS-SF in Hebrew for other groups (e.g., Israeli Jews vs Israeli Arabs).

Beyond measurement invariance, it is also important to consider how the FEVS and FEVS-SF relate to key sociodemographic characteristics across contexts. In this study, we conducted an exploratory investigation of associations between FEVS-SF factors and income and indeed found that higher income was associated with lower scores on both factors, but more so for the well-being factor. Other factors such as age, gender, and education may also relate to the FEVS and FEVS-SF, as has been seen in studies conducted within the United States (e.g., Lichtenberg et al., 2020, 2021; Moray & Lichtenberg, 2025). In comparing those studies to the present study, we find some differences in relationships to these key characteristics. For example, while we did not find significant associations with age and the FEVS or FEVS-SF, these associations were reported in some of the US studies (e.g., Lichtenberg et al., 2020) but not all (e.g., Lichtenberg et al., 2021). Divergence between studies may arise due to sampling differences (online samples as in Lichtenberg et al., 2021 and ours; vs participants of SAFE program as in Lichtenberg et al. 2020; Moray & Lichtenberg, 2025) as well as sample size differences. This underscores the need for future research that specifically explores contextual and sampling variations that may influence FEVS and FEVS-SF assessments.

Taken together, the FEVS-SF is a short 9-item assessment that may be of great benefit to researchers and clinicians who are limited in time and resources. The FEVS short-form has the potential to provide a solution to FE researchers by overcoming some of the inherent challenges in assessing FE experiences in older adulthood. These challenges include the underreporting of FE for various reasons, including embarrassment and fear, and the low statistical power often associated with assessing experienced FE due to its low base rate in the population. Of course, we do not recommend that risk measures replace assessments of actual FE experiences, as one’s vulnerability level will not necessarily align perfectly with one’s actual experiences. Importantly, future studies should consider the FEVS-SF as a standalone measure, as in this study participants responded to the full 17-item measure from which the short-form scores were then derived. Certain items not included in the short form may have influenced responses on items considered within the short-form. Another important avenue of future research is to further explore the two conceptual factors identified, financial well-being, and financial decision making, within the FEVS-SF. Considering each of these subscales separately may provide additional utility in understanding factors that contribute to FE vulnerability. Finally, future studies should examine other forms of reliability and validity including criterion validity by examining the measure’s alignment with confirmed cases of FE. This may further enhance the clinical utility of this measure.

Despite this study’s notable strength of using a large pooled sample to examine the measurement property evidence of the FEVS and FEVS-SF, there are also several limitations that should be mentioned. First, the four samples included in the present study were recruited using convenience-sampling techniques, thereby limiting representativeness of the full combined sample to the Israeli population and other non-Israel populations. Also, because of the way data was collected, that is, by different students over four years approaching older adults to take part, there is a small chance that some participants may have participated in more than one of the four studies incorporated in the pooled dataset. As the data were collected anonymously this cannot be verified. Nonetheless, we consider the likelihood of repeat participants in the pooled dataset to be low. Each year, recruitment was carried out by a new cohort of students who approached older adults within their own social circles or community organizations, making overlap unlikely. Nevertheless, to address this limitation, we also conducted analyses separately for each study in order to compare results against the pooled sample. A further limitation is that data across the four studies were collected online thereby excluding older adults who do not have access to computers or the internet and introducing a potential bias in the sample to those with technology access or higher literacy. Our participant recruitment and data collection procedures may have also contributed to the relatively high rate of FE reported in our datasets and in the amalgamated sample. Prevalence rates of FE vary widely in the literature, largely due to differences in how FE is defined and methodological approaches (Jackson, 2018). Importantly, the datasets were not based on representative population-based samples and therefore the observed rates of FE should not be interpreted as an estimate of population prevalence. Nevertheless, the relatively high rate of self-reported FE across datasets (19%–23%) may reflect the anonymous online format of the studies. Such a format may eliminate barriers to FE disclosure such as fear and shame that are commonly seen in non-anonymized investigations (Jackson & Hafemeister, 2013; Lachs & Berman, 2011). A final notable limitation relates to the cultural sensitivity of the FEVS and FEVS-SF. While translation and back-translation methods were implemented and consensus meetings resolved discrepancies, we note that further validation of participants’ understanding of items (e.g., via direct questions or cognitive testing) was not implemented in any of the studies. Future validation studies are warranted to examine cultural sensitivity of the measures.

Nevertheless, findings of this study support use of the FEVS-SF in Hebrew to measure FE vulnerability, and specifically point to two factors that may provide meaningful information regarding the overarching construct of FE vulnerability. The FEVS-SF is a measure that can be administered easily and at low cost, and may serve useful to clinicians and researchers alike. While this study focused on the Hebrew version of the FEVS and FEVS-SF, implications of the findings extend beyond use of the measures in Hebrew. Future studies may consider expanding upon the validity of the FEVS in Hebrew, and examining its utility in other languages.

## Supplementary Material

gnag050_Supplementary_Data

## Data Availability

The data, analytic methods, and materials can be made available to other researchers upon reasonable request to the corresponding author. The studies reported in this manuscript were not pre-registered.
