# Supplementary material for: Evaluating the Hebrew version of the financial exploitation vulnerability scale
Source: Gerontologist. 2026 Apr 17;66(6):gnag050. doi: 10.1093/geront/gnag050 (PMC13098708; doi:10.1093/geront/gnag050)
Supplement: gnag050_Supplementary_Data [file gnag050_supplementary_data.zip › Weissberger_etal_Supplementary_Tables.docx]

**Evaluating the Hebrew version of the Financial Exploitation Vulnerability Scale**

**Gali H. Weissberger^1*^, PhD, Lisa Engel^2,3^, PhD, S. Duke Han^4^, PhD, Peter Lichtenberg^5^, PhD**

^1^Department of Social and Health Sciences, Bar-Ilan University, Ramat Gan, Israel, Gali.Weissberger@biu.ac.il

ORCID ID: 0000-0002-3648-4866

^2^ Department of Occupational Therapy, College of Rehabilitation Sciences, University of Manitoba, Winnipeg, MB, Canada

Lisa.Engel@umanitoba.ca

ORCID ID: 0000-0002-3208-9850

^3^Institute for Work and Health, Toronto, Ontario, Canada

^4^Department of Psychology, USC Dornsife College of Letters, Arts, and Sciences, Los Angeles, CA, USA

dukehan@usc.edu

ORCID ID: 0000-0003-2453-9230

^5^Institute of Gerontology, Wayne State University, Detroit, Michigan, USA

p.lichtenberg@wayne.edu

ORCID ID: 0000-0001-6632-6714

*Corresponding author

**Supplementary Table 1.** Bivariate associations between background characteristics and the FEVS/FEVS-SF.

| **Dataset** | **Measure** | **Bivariate Associations** | | | | | | | | | | | | | |
| --- | --- | --- | --- | --- | --- | --- | --- | --- | --- | --- | --- | --- | --- | --- | --- |
|  |  | **Age** | |  | **Sex** | |  | **Education** | |  | **Income** | |  | **Sum of Illnesses** | |
|  |  | *r* | *p*-value |  | *t* | *p*-value |  | *r* | *p*-value |  | *r* | *p*-value |  | *r* | *p*-value |
| 1 | FEVS | 0.02 | >0.999 |  | -1.95 | 0.053 |  | -0.21 | 0.18 |  | -0.26 | 0.06 |  | 0.16 | 0.492 |
|  | FEVS-SF | -0.03 | >0.999 |  | -2.38 | 0.019 |  | -0.20 | 0.180 |  | -0.30 | 0.014 |  | 0.16 | 0.492 |
| 2 | FEVS | <0.01 | >0.999 |  | -2.23 | 0.028 |  | -0.23 | 0.041 |  | -0.43 | <0.001 |  | -0.43 | <0.001 |
|  | FEVS-SF | -0.05 | >0.999 |  | -2.32 | 0.022 |  | -0.19 | 0.096 |  | -0.40 | <0.001 |  | 0.12 | 0.422 |
| 3 | FEVS | 0.04 | 0.555 |  | -0.35 | 0.726 |  | -0.27 | <0.001 |  | -0.34 | <0.001 |  | 0.24 | <0.001 |
|  | FEVS-SF | <0.01 | 0.847 |  | -1.76 | 0.078 |  | -0.28 | <0.001 |  | -0.34 | <0.001 |  | 0.19 | <0.001 |
| 4 | FEVS | -0.07 | 0.507 |  | 0.38 | 0.704 |  | -0.14 | 0.043 |  | -0.35 | <0.001 |  | 0.19 | 0.004 |
|  | FEVS-SF | -0.11 | 0.128 |  | -0.12 | 0.908 |  | -0.12 | 0.128 |  | -0.35 | <0.001 |  | 0.14 | 0.043 |

Note: FEVS = Financial Exploitation Vulnerability Scale; FEVS-SF = Financial Exploitation Vulnerability Scale, Short Form

**Supplementary Table 2.** Standardized factor loadings of the confirmatory factor analyses for the FEVS and FEVS-SF.

|  | **a. FEVS** | **b. FEVS-SF** |
| --- | --- | --- |
|  | **Standardized factor loading** | **Standardized factor loading** |
| Item 1 | 0.707 | 0.720 |
| Item 2 | 0.666 | 0.670 |
| Item 3 | 0.083 | - |
| Item 4 | 0.537 | 0.522 |
| Item 5 | 0.428 | 0.413 |
| Item 6 | 0.656 | 0.661 |
| Item 7 | 0.112 | - |
| Item 8 | 0.502 | 0.511 |
| Item 9 | 0.139 | - |
| Item 10 | 0.225 | 0.224 |
| Item 11 | 0.674 | 0.677 |
| Item 12 | 0.201 | - |
| Item 13 | 0.742 | 0.748 |
| Item 14 | 0.296 | - |
| Item 15 | 0.326 | - |
| Item 16 | 0.240 | - |
| Item 17 | 0.340 | - |

Note: FEVS = Financial Exploitation Vulnerability Scale; FEVS-SF = Financial Exploitation Vulnerability Scale, Short Form

**Supplementary Table 3.** Factor loadings for the two-factor solution of the FEVS.

|  | **Item** | **Factor 1** | **Factor 2** |
| --- | --- | --- | --- |
| FEVS 1 | How worried are you about having enough money to pay for things? | 0.703 | -0.185 |
| FEVS 2 | Overall, how satisfied are you with your finances? | 0.663 | -0.121 |
| FEVS 3 | Who manages your money day to day? |  | 0.124 |
| FEVS 4 | How satisfied are you with this (money management) arrangement? | 0.535 |  |
| FEVS 5 | How confident are you in making big financial decisions? | 0.426 |  |
| FEVS 6 | How often do you worry about financial decisions you've recently made? | 0.647 | -0.11 |
| FEVS 7 | Have you noticed any money taken from your bank account without your permission? | 0.117 |  |
| FEVS 8 | How often do your monthly expenses exceed your regular monthly income? | 0.496 | -0.14 |
| FEVS 9 | How often do you talk with or visit others on a regular basis? | 0.138 |  |
| FEVS 10 | How often do you wish you had someone to talk to about financial decisions, transactions, or plans? | 0.222 |  |
| FEVS 11 | How often do you feel anxious about your financial decisions and/or transactions? | 0.664 |  |
| FEVS 12 | Do you have a confidante with whom you can discuss anything, including your financial situation and decisions? | 0.202 |  |
| FEVS 13 | How often do you feel downhearted or blue about your financial situation or decisions? | 0.738 | -0.124 |
| FEVS 14 | Are your memory, thinking skills, or ability to reason with regard to financial decisions or financial transactions worse than a year ago? | 0.304 | 0.138 |
| FEVS 15 | Has a relationship with a family member or friend become strained due to finances as you have gotten older? | 0.353 | 0.319 |
| FEVS 16 | Did anyone ever tell you that someone else you know wants to take your money? | 0.284 | 0.504 |
| FEVS 17 | How likely is it that anyone now wants to take your money without your permission? | 0.403 | 0.599 |

Note: FEVS = Financial Exploitation Vulnerability Scale

**Supplementary Table 4.** Factor loadings for the three-factor solution of the FEVS.

|  | **Item** | **Factor 1** | **Factor 2** | **Factor 3** |
| --- | --- | --- | --- | --- |
| FEVS 1 | How worried are you about having enough money to pay for things? | 0.726 | 0.120 | 0.122 |
| FEVS 2 | Overall, how satisfied are you with your finances? | 0.675 |  | 0.174 |
| FEVS 3 | Who manages your money day to day? | -0.121 | 0.453 |  |
| FEVS 4 | How satisfied are you with this (money management) arrangement? | 0.424 | 0.290 | 0.182 |
| FEVS 5 | How confident are you in making big financial decisions? | 0.185 | 0.662 | 0.113 |
| FEVS 6 | How often do you worry about financial decisions you've recently made? | 0.558 | 0.352 | 0.109 |
| FEVS 7 | Have you noticed any money taken from your bank account without your permission? |  |  | 0.125 |
| FEVS 8 | How often do your monthly expenses exceed your regular monthly income? | 0.517 |  |  |
| FEVS 9 | How often do you talk with or visit others on a regular basis? | 0.150 |  |  |
| FEVS 10 | How often do you wish you had someone to talk to about financial decisions, transactions, or plans? | 0.103 | 0.337 |  |
| FEVS 11 | How often do you feel anxious about your financial decisions and/or transactions? | 0.557 | 0.392 | 0.118 |
| FEVS 12 | Do you have a confidante with whom you can discuss anything, including your financial situation and decisions? | 0.205 |  | 0.121 |
| FEVS 13 | How often do you feel downhearted or blue about your financial situation or decisions? | 0.705 | 0.189 | 0.180 |
| FEVS 14 | Are your memory, thinking skills, or ability to reason with regard to financial decisions or financial transactions worse than a year ago? | 0.157 | 0.249 | 0.208 |
| FEVS 15 | Has a relationship with a family member or friend become strained due to finances as you have gotten older? | 0.188 |  | 0.430 |
| FEVS 16 | Did anyone ever tell you that someone else you know wants to take your money? |  |  | 0.571 |
| FEVS 17 | How likely is it that anyone now wants to take your money without your permission? | 0.111 | 0.117 | 0.721 |

Note: FEVS = Financial Exploitation Vulnerability Scale

**Supplementary Table 5.** Factor loadings for the four-factor solution of the FEVS.

|  | **Item** | **Factor 1** | **Factor 2** | **Factor 3** | **Factor 4** |
| --- | --- | --- | --- | --- | --- |
| FEVS 1 | How worried are you about having enough money to pay for things? | 0.808 |  |  |  |
| FEVS 2 | Overall, how satisfied are you with your finances? | 0.673 |  |  |  |
| FEVS 3 | Who manages your money day to day? |  |  | 0.520 | -0.217 |
| FEVS 4 | How satisfied are you with this (money management) arrangement? | 0.161 |  | 0.188 | 0.470 |
| FEVS 5 | How confident are you in making big financial decisions? |  |  | 0.649 | 0.135 |
| FEVS 6 | How often do you worry about financial decisions you've recently made? | 0.524 |  | 0.216 |  |
| FEVS 7 | Have you noticed any money taken from your bank account without your permission? |  | 0.117 |  |  |
| FEVS 8 | How often do your monthly expenses exceed your regular monthly income? | 0.574 |  |  |  |
| FEVS 9 | How often do you talk with or visit others on a regular basis? |  |  |  | 0.170 |
| FEVS 10 | How often do you wish you had someone to talk to about financial decisions, transactions, or plans? | 0.137 |  | 0.320 | -0.104 |
| FEVS 11 | How often do you feel anxious about your financial decisions and/or transactions? | 0.524 |  | 0.253 |  |
| FEVS 12 | Do you have a confidante with whom you can discuss anything, including your financial situation and decisions? |  |  | -0.138 | 0.459 |
| FEVS 13 | How often do you feel downhearted or blue about your financial situation or decisions? | 0.708 |  |  |  |
| FEVS 14 | Are your memory, thinking skills, or ability to reason with regard to financial decisions or financial transactions worse than a year ago? | 0.124 | 0.175 | 0.197 |  |
| FEVS 15 | Has a relationship with a family member or friend become strained due to finances as you have gotten older? |  | 0.408 |  | 0.137 |
| FEVS 16 | Did anyone ever tell you that someone else you know wants to take your money? |  | 0.617 |  |  |
| FEVS 17 | How likely is it that anyone now wants to take your money without your permission? |  | 0.716 |  |  |

Note: FEVS = Financial Exploitation Vulnerability Scale

**Supplementary Table 6.** Model comparisons for testing measurement invariance across sex, income, and education groups.

|  | **Sex** | |  | **Income** | |  | **Education** | |
| --- | --- | --- | --- | --- | --- | --- | --- | --- |
| **Model** | **ΔCFI (CFI)** | **ΔRMSEA (RMSEA)** |  | **ΔCFI (CFI)** | **ΔRMSEA (RMSEA)** |  | **ΔCFI (CFI)** | **ΔRMSEA (RMSEA)** |
| Configural invariance | (.995) | (.044) |  | (.992) | (.048) |  | (.994) | (.046) |
| Metric invariance (loadings) | -.001 | .001 |  | <.001 | -.003 |  | .001 | .001 |
| Scalar invariance (loadings and intercepts) | <.001 | -.005 |  | -.001 | <.001 |  | .001 | -.005 |
| Strict invariance (loadings, intercepts, and residual variances) | <.001 | <.001 |  | <.001 | <.001 |  | <.001 | <.001 |

**Appendix A.** Hebrew version of the FEVS

**שאלון סולם פגיעות לניצול פיננסי – Financial Exploitation Vulnerability Scale**

**נושא מערך שאלות זה הוא חווייתך בעניינים פיננסים ובהחלטות פיננסיות וכיצד את/ה ואחרים מתנהגים בנוגע לעניינים פיננסים ולהחלטות פיננסיות. אנא בחרו תשובה אחת בלבד לכל שאלה.**

1. עד כמה את/ה מודאג לגבי האם יש ברשותך מספיק כסף כדי לשלם עבור צרכייך?

בכלל לא מודאג (0)

קצת מודאג (1)

מאוד מודאג (2)

2. בסך הכול, עד כמה את/ה מרוצה ממצבך הכספי?

מרוצה (0)

לא מרוצה אך גם לא בלתי מרוצה (1)

בלתי מרוצה (2)

3. מי מנהל את כספך?

אני, ללא שום עזרה (0)

אני מקבל/ת עזרה ממישהו (1)

מישהו אחר מנהל את כספי (2)

4. עד כמה את/ה מרוצה מהסידור (ניהול כספי) הזה?

מרוצה (0)

לא מרוצה אך גם לא בלתי מרוצה (1)

בלתי מרוצה (2)

5. עד כמה את/ה בטוח/ה בעצמך בקבלת החלטות כספיות גדולות?

בטוח בעצמי (0)

לא בטוח בעצמי אך גם לא בלתי בטוח בעצמי (1)

בלתי בטוח בעצמי (2)

6. באיזה תדירות את/ה מודאג מהחלטות כספיות שקיבלת לאחרונה?

אינני דואג (0)

לפעמים (1)

לעתים קרובות (2)

7. האם הבחנת שכסף הוצא מהחשבון הבנק שלך ללא רשותך?

לא (0)

כן (1)

8. באיזה תדירות ההוצאות החודשיות עולות על ההכנסות החודשיות הרגילות שלך?

לעתים נדירות או אף פעם (0)

חלק מהזמן (1)

רוב הזמן (2)

9. באיזה תדירות את/ה מדבר/ת או מבקר/ת אחרים?

יומי או שבועי (0)

חודשי (1)

פחות מחודשי (2)

10. באיזה תדירות היית רוצה שיהיה לך עם מי לדבר לגבי החלטות, פעולות, או תוכניות כספיות?

אף פעם (0)

חלק מהזמן (1)

רוב הזמן (2)

11. באיזה תדירות את/ה מרגיש חרד/ה לגבי ההחלטות והפעולות הכספיות שלך?

לעתים נדירות או אף פעם (0)

לפעמים (1)

לעתים קרובות (2)

12. האם יש לך איש קרוב שעמו את/ה יכול לדון בכל נושא, כולל מצבים והחלטות כספיות?

כן (0)

לא (1)

13. באיזה תדירות את/ה מרגיש מיואש/ת או עצוב/ה לגבי המצב הכספי או ההחלטות הכספיות שלך?

אף פעם (0)

חלק מהזמן (1)

רוב הזמן (2)

14. האם הזיכרון, כושר המחשבה, או יכולתך לנתח החלטות ופעולות כספיות טובים פחות היום ביחס לשנה שעברה?

לא (0)

כן (1)

15. האם מערכת יחסים עם בן משפחה או חבר הפכה למתוחה בשל עניינים כספיים כאשר הזדקנת?

לא (0)

כן (1)

16. האם מישהו אמר לך שמישהו אחר רוצה לקחת את כספך?

לא (0)

כן (1)

17. מהי הסבירות שמישהו עכשיו רוצה לקחת או להשתמש בכספך ללא רשותך?

בלתי סביר (0)

סביר במידה מועטה (1)

סביר במידה רבה (2)
