# Supplementary figures and images for: Evaluating the Hebrew version of the financial exploitation vulnerability scale
Source: Gerontologist. 2026 Apr 17;66(6):gnag050. doi: 10.1093/geront/gnag050 (PMC13098708; doi:10.1093/geront/gnag050)

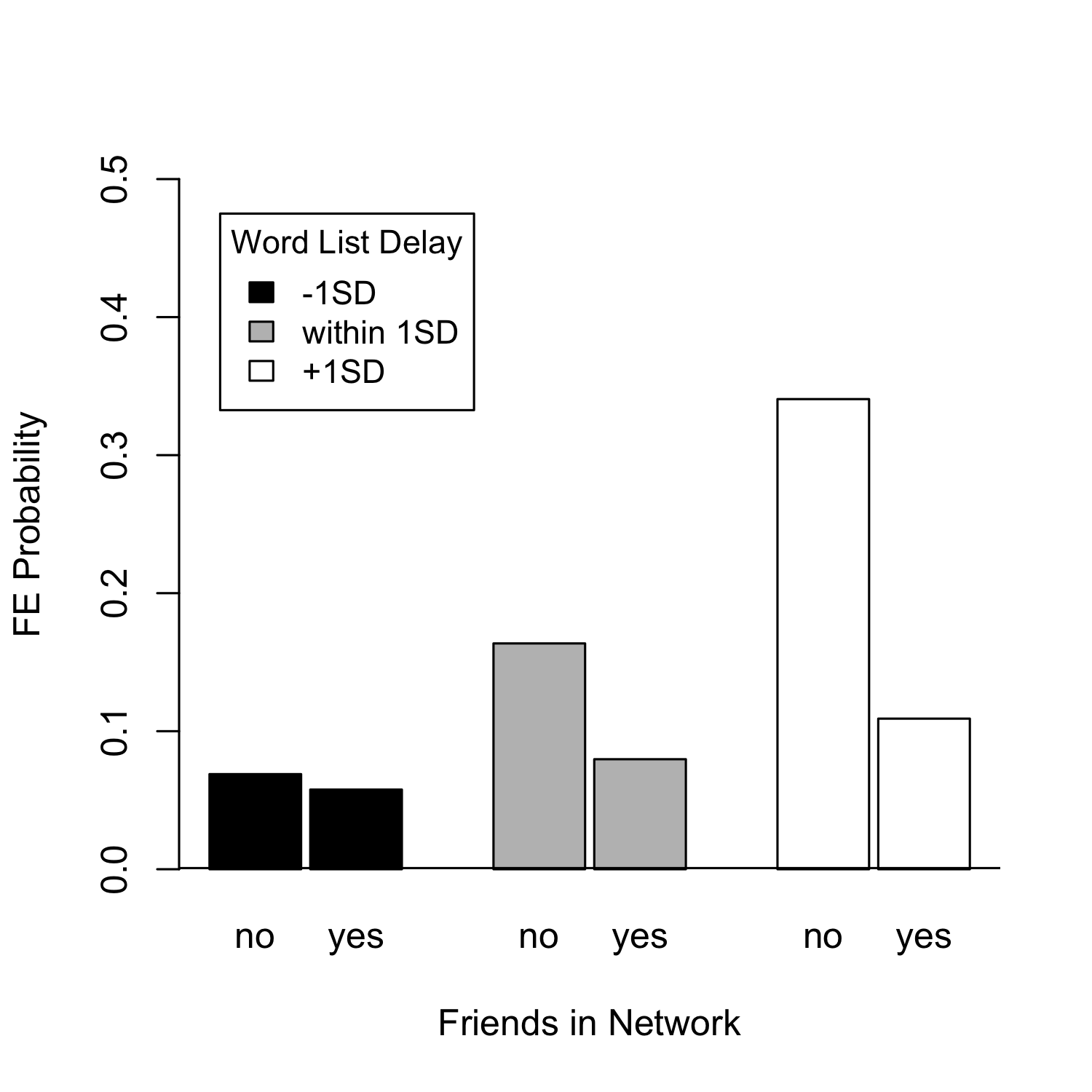

Supplement: gnag050_Supplementary_Data [file gnag050_supplementary_data.zip › figure1_supp_highres.tiff]
